# Correction to “Expression and Function of Meflin in Human Endometrial Decidualization”

**DOI:** 10.1002/rmb2.70054

**Published:** 2026-04-26

N. Inoue, Y. Tsukui, A. Morita, et.al., Expression and Function of Meflin in Human Endometrial Decidualization. *Reproductive Medicine and Biology* 25, no. 1 (2026): e70048.

In Reference 33, the journal name was published as Biomedicines; this should read *Biomedicine**s***.

In Reference 39, the title, journal, and pagination, “Aberrant expression of deoxyribonucleic acid methyltransferases **and Methylation of Progesterone Receptor Gene in** Endometriosis, **American Journal of Obstetrics and Gynecology 194, no. 4 (2006): 1104–1110**.” were incorrect. This should have read: Aberrant Expression of Deoxyribonucleic Acid Methyltransferases **DNMT1, DNMT3A, and DNMT3B in Women With Endometriosis. *Fertility and Sterility* 87, no. 1 (2007): 24–32**.

In Reference 40, the author, title, journal name, and pagination, “Yin X., Pavone ME., Lu Z., Wei J., and Kim **J**, Increased Activation of the PI3K/AKT Pathway Compromises Decidualization of Stromal Cells **Derived From Endometriotic Endometrium**, **Fertility and Sterility 97, no. 5 (2012): 1128–1135.e3**” were incorrect. This should have read: X. Yin, M. E. Pavone, Z. Lu, **J. J**. Wei, J. J. Kim. Increased Activation of the PI3K/AKT Pathway Compromises Decidualization of Stromal Cells **From Endometriosis. *Journal of Clinical Endocrinology Metabolism* 97, no. 1 (2012): 35–43**.

In Reference 42, the authors, title, journal, and pagination, “Brown DN., **Fan Y., Choi Y., et al**., Notch1 Signaling **Regulates Progesterone Receptor Expression and Decidualization in Human Endometrial Stromal Cells**, **Journal of Clinical Endocrinology and Metabolism 103, no. 5 (2018): 1912–1923**.” were incorrect. This should have read: D. M. Brown, **H. C. Lee, S. Liu, et al**., Notch‐1 Signaling **Activation and Progesterone Receptor Expression in Ectopic Lesions of Women With Endometriosis. *Journal of the Endocrine Society* 2, no. 7 (2018): 765–778**.

In Reference 43, the title, journal, and pagination, “**M**iR‐194‐3p **Regulates Progesterone Responsiveness via Targeting Progesterone Receptor in Endometrial Stromal Cells, Cellular Physiology and Biochemistry 45, no. 6 (2018): 2267–2279**.” were incorrect. This should have read: **m**iR‐194‐3p **Represses the Progesterone Receptor and Decidualization in Eutopic Endometrium From Women With Endometriosis. *Endocrinology* 159, no, 7 (2018): 2554–2562**.

We apologize for these errors.

